# A Relação entre o Estado Dipping Noturno, Aumento da Pressão Arterial Matinal e Internações Hospitalares em Pacientes com a Insuficiência Cardíaca Sistólica

**DOI:** 10.36660/abc.20220932

**Published:** 2023-09-04

**Authors:** Ümmü Taş, Sedat Taş, Efe Edem

**Affiliations:** 1 Izmir Demokrasi Universitesi Karabaglar Turquia Izmir Demokrasi Universitesi – Cardiology, Karabaglar – Turquia; 2 Manisa Celal Bayar University Manisa Turquia Manisa Celal Bayar University – Cardiology, Manisa – Turquia; 3 İzmir Tınaztepe University İzmir Turquia İzmir Tınaztepe University – Cardiology, İzmir – Turquia

**Keywords:** Monitorização Ambulatorial da Pressão Arterial, Pressão Arterial, Insuficiência Cardíaca, Hospitalização

## Abstract

**Fundamento:**

A hipertensão é um fator de risco conhecido para o desenvolvimento de insuficiência cardíaca. No entanto, há dados limitados para investigar a associação entre pico de pressão arterial matinal (PPAM), estado dipper, parâmetros ecocardiográficos e internações hospitalares em pacientes com insuficiência cardíaca sistólica.

**Objetivos:**

Avaliar a relação entre aumento matinal da pressão arterial, padrão de pressão arterial não-dipper, parâmetros ecocardiográficos e internações hospitalares em pacientes com insuficiência cardíaca sistólica.

**Métodos:**

Analisamos retrospectivamente os dados de 206 pacientes consecutivos com hipertensão e fração de ejeção do ventrículo esquerdo abaixo de 40%. Dividimos os pacientes em dois grupos de acordo com os resultados da monitoramento ambulatorial da pressão arterial (MAPA) de 24 horas: dippers (n=110) e não-dippers (n=96). O aumento matinal da pressão arterial foi calculado. Achados ecocardiográficos e internações hospitalares durante o acompanhamento foram anotados. A significância estatística foi definida como p < 0,05.

**Resultados:**

O grupo de estudo foi composto por 206 pacientes com predominância do sexo masculino e idade média de 63,5 ± 16,1 anos. O grupo não-dipper teve significativamente mais internações hospitalares em comparação com os dippers. Houve correlação positiva entre PPAM e índice de volume do átrio esquerdo (r=0,331, p=0,001), relação entre velocidade de influxo mitral precoce e velocidade de propagação do fluxo (r= 0,326, p=0,001) e relação entre influxo mitral precoce velocidade e velocidade diastólica inicial do anel mitral (E/Em) (r= 0,314, p=0,001). Verificou-se que a PA não-dipper, PPAM e o padrão E/Em estão independentemente associados ao aumento das admissões hospitalares.

**Conclusão:**

O PPAM está associado à disfunção diastólica e pode ser um preditor sensível de internação hospitalar em pacientes com insuficiência cardíaca sistólica.

**Figura Central:**
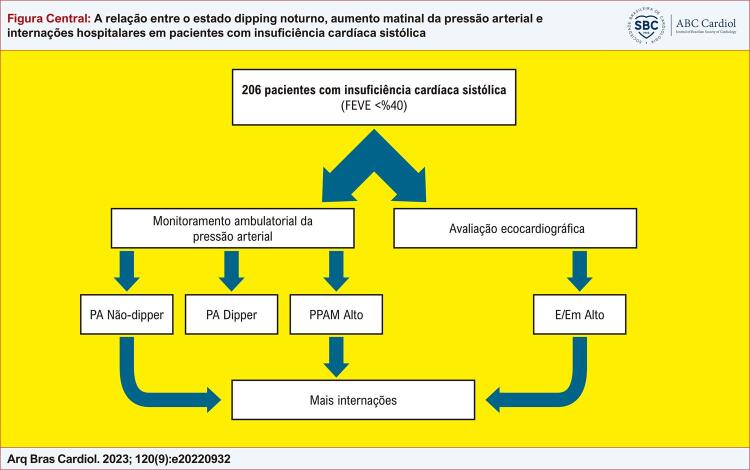
: A relação entre o estado dipping noturno, aumento matinal da pressão arterial e internações hospitalares em pacientes com insuficiência cardíaca sistólica

PA: Pressão arterial; PPAM: Aumento matinal da pressão arterial; E/Em: Razão entre o pico precoce (E) da velocidade de influxo mitral e a velocidade diastólica precoce do anel mitral (Em).

## Introdução

A hipertensão (HA) está entre os fatores de risco cardiovasculares mais tratáveis. Observações sobre variações circadianas na PA levaram a uma nova classificação em HA. O’Brien et al. chamou a atenção para o significado prognóstico da redução noturna da pressão arterial (PA) e propôs pela primeira vez os conceitos de ‘dippers’ e ‘não-dippers’.^1^ Dippers foram definidos como pessoas cuja PA noturna diminuiu em 10% ou mais do que os valores diurnos, enquanto aqueles cuja PA noturna diminuiu menos de 10% são não-dippers. Como a PA já diminui à noite com o ritmo circadiano normal, a redução da PA noturna em pacientes com HA é definida como um bom fator de prognóstico. Assim, o monitoramento ambulatorial da pressão arterial de 24 horas (MAPA de 24 horas) para identificar padrões dipping ou não-dipping tornou-se cada vez mais importante para o manejo de pacientes com HA.^2^

Parâmetros cardiovasculares, como PA, tônus coronário e frequência cardíaca, mudam com o ritmo circadiano.^3^ Fox e Mulcahy mostraram que as variações circadianas na frequência cardíaca e na pressão arterial eram virtualmente idênticas em indivíduos normotensos; ambas caíram e permaneceram relativamente baixas durante toda a noite e depois subiram acentuadamente nas primeiras horas da manhã para atingir um pico durante a manhã.^4^ Há um interesse crescente no papel da HA não-dipping e do aumento matinal da PA (PPAM) em várias doenças cardiovasculares e cerebrovasculares, incluindo disfunção diastólica ventricular esquerda e direita, hipertrofia ventricular esquerda, infarto do miocárdio, insuficiência cardíaca e acidente vascular cerebral.^5,6^ A influência da hipertensão arterial na remodelação estrutural e funcional do ventrículo esquerdo é bem conhecida.^7,8^ No entanto, os efeitos da PPAM e o padrão não-dipper na função diastólica e internações hospitalares em pacientes com insuficiência cardíaca com fração de ejeção reduzida (ICFEr) são pouco descritos.

A insuficiência cardíaca é um importante problema de saúde pública devido à sua associação com morbidade significativa, internações hospitalares recorrentes, aumento dos custos de saúde e mortalidade.^9,10^ Em seu estudo, Mosalpuria et al. analisaram dados de consultas ambulatoriais de pacientes com insuficiência cardíaca. Eles encontraram a HA como a comorbidade mais comum (62%) e sugeriram que um melhor tratamento ambulatorial poderia melhorar os resultados e reduzir o número de reinternações.^11^ Avaliar o papel da variação circadiana da PA na insuficiência cardíaca pode esclarecer a eficácia do tratamento e o momento de dosagem de medicamentos em casos individuais. A internação hospitalar, principalmente recorrente, por insuficiência cardíaca é um importante fator de risco para mortalidade. Portanto, determinar os fatores de risco de internação hospitalar e preveni-los pode ser um método considerável e custo-efetivo para reduzir a mortalidade por IC. Este estudo foi conduzido para determinar os efeitos da variabilidade circadiana da PA na admissão hospitalar em pacientes com ICFEr e analisar as relações entre o declínio noturno da PA, PPAM e internação hospitalar.

## Métodos

### Populações de estudo

Este estudo de caso-controle retrospectivo foi projetado e conduzido de acordo com a Declaração de Helsinque e aprovado por um comitê de ética institucional da Universidade İzmir Tınaztepe (Data e número da decisão: 2021/13). Coletamos os achados de MAPA e ecocardiograma de 224 pacientes com insuficiência cardíaca sistólica que se apresentaram nos ambulatórios de cardiologia de nosso hospital entre novembro de 2018 e dezembro de 2019 e foram diagnosticados ou recém-diagnosticados com HA com base na avaliação inicial do ambulatório e, em seguida, realizamos monitoramento ambulatorial da pressão arterial por 24 horas. Também coletamos internações hospitalares de cada paciente desde a avaliação inicial até 2 anos de acompanhamento. Dos 224 pacientes, excluímos aqueles perdidos no seguimento (n = 18). Dados referentes às internações do paciente durante o acompanhamento, histórico médico, e informações sociodemográficas e clínicas foram obtidas de seus prontuários. O índice de massa corporal foi anotado para cada paciente (kg/m^2^).

Definimos insuficiência cardíaca sistólica e diastólica de acordo com as diretrizes da ESC de 2021 para diagnóstico e tratamento de insuficiência cardíaca aguda e crônica.^12,13^ ICFEr foi definida como uma fração de ejeção do ventrículo esquerdo inferior a 40%. Admissões hospitalares durante o seguimento foram identificadas para todos os pacientes. Avaliamos todas as internações por insuficiência cardíaca sintomática 2 anos após a avaliação inicial.

### Monitoramento ambulatorial da pressão arterial

Definimos HA como PA sistólica superior a 140 mmHg e/ou PA diastólica superior a 90 mmHg, de acordo com os critérios especificados nas Diretrizes da *European Society of Hypertension/European Society of Cardiology (ESC)*^12^ para o manejo da hipertensão arterial. Para este estudo, calculamos a PA ambulatorial diurna como a PA média das 6h às 20h e a PA noturna como a PA média entre as 20h e as 6h. Também determinamos os valores médios da PA sistólica e diastólica de 24 horas, diurna e noturna e calculamos o PPAM subtraindo a PA sistólica noturna mínima da PA sistólica média nas primeiras 2 horas após levantar-se. Com base nos resultados da MAPA de 24 horas, os pacientes foram divididos em grupo dipper (redução ≥10% na PA noturna) e não-dipper (redução <10% na PA noturna).

### Ecocardiografia

Diâmetros sistólicos e diastólicos do ventrículo esquerdo, índice de volume do átrio esquerdo, diâmetro aórtico, fração de ejeção do ventrículo esquerdo (método de Simpson), velocidades E e A do influxo mitral, tempo de desaceleração, tempo de relaxamento isovolumétrico, relação entre precoce (E) e tardio (A) pico da velocidade de entrada mitral, a relação entre a velocidade de influxo mitral inicial e a velocidade diastólica inicial do anel mitral (E/Em) e os achados do Doppler tecidual avaliados por ecocardiografia bidimensional obtida de eixos paraesternais longos e curtos padrão e apical de 2, 3 e 4 câmaras opiniões foram coletadas.

### Análise estatística

Os dados foram analisados no programa SPSS versão 15.0 (SPSS Inc, Chicago, EUA). O teste de normalidade foi realizado por meio do teste de Kolmogorov-Smirnov. Dados categóricos são expressos como contagem e porcentagem, e dados contínuos como média e desvio padrão (DP). Os dados categóricos foram comparados usando o teste qui-quadrado, e os dados contínuos foram comparados usando um teste t independente. Os coeficientes de correlação de Pearson (r) foram usados para examinar as correlações entre o PPAM e o peptídeo natriurético cerebral, valores de PA e achados ecocardiográficos. Uma correlação parcial foi realizada para controlar o efeito dos fatores de confusão, incluindo sexo e idade. Fatores com significância estatística (p < 0,05) foram incluídos na análise de regressão logística para determinar preditores independentes de internação. Um procedimento de seleção de variáveis foi implementado, e todas essas variáveis candidatas foram alimentadas nos procedimentos de seleção direta e eliminação regressiva, além de forçar todas as variáveis do modelo juntas. Análises multivariadas de riscos proporcionais de Cox usando o método Enter foram calculadas para identificar os preditores independentes de mortalidade. A análise da curva característica de operação do receptor das variáveis selecionadas foi feita para detectar os valores de corte com maior sensibilidade e especificidade na previsão de internações hospitalares. A significância estatística foi definida como p < 0,05.

## Resultados

### Linha de base e características clínicas

O grupo de estudo compreendeu 206 pacientes com predominância do sexo masculino (84 [40,8%] mulheres, 122 [59,2%] homens) e idade média de 63,5 ± 16,1 (24–94) anos. De acordo com os resultados do MAPA de 24 horas, 110 pacientes (53,3%) eram dippers e 96 pacientes (46,7%) eram não-dippers. A classe da New York Heart Association (NYHA) foi II em 54,4%, III em 38,8% e IV em 6,8% dos pacientes. Durante o período do estudo, 206 pacientes tiveram 556 internações por insuficiência cardíaca confirmada (1,34 internações por paciente por ano). O grupo não-dipper teve uma taxa de admissão hospitalar significativamente maior do que o grupo dipper (p <0,001). Não houve diferenças significativas entre os grupos em comorbidades, incluindo diabetes mellitus, doença renal crônica, doença cardíaca isquêmica crônica, doença pulmonar obstrutiva crônica e hiperlipidemia, ou termos das drogas utilizadas pelos pacientes, exceto diuréticos. As características clínicas dos pacientes estão resumidas na Tabela 1.

**Tabela 1:** – Características clínicas e demográficas basais

	Dipper (n:110)	Não-dipper (n:96)	valor-p
Genero masculino (%)	68(61.8)	54(56.3)	0,566^¥^
Idade (anos)	57,0±16,3	60,1±15,8	0,325*
**NYHA FC, n (%)**			0,094^¥^
FC-2	70(63.6)	42(43.8)	
FC-3	36(32.7)	44(45.8)	
FC-4	4(3.6)	10(10.4)	
Número de internações (ano)	1,01±0,59	1,72±0,81	<0,001^*^
Tabagismo, n (%)	16(14.5)	16(16.7)	0,767^¥^
DM, n (%)	34(30.9)	32(33.3)	0,793^¥^
DRC, n (%)	10(9.1)	10(10.4)	0,821^¥^
HL, n (%)	22(20.0)	24(25,0)	0,543^¥^
DCIC, n (%)	72(65,5)	74(77.1)	0,195^¥^
DPOC, n (%)	6(5.5)	16(16.7)	0,066^¥^
Diurético, n (%)	82(74,5)	94(94,9)	**0,001**^**¥**^
IECA-BRA, n (%)	94(85,5)	84(87,5)	0,806^¥^
Espironalactona, n (%)	76(69.1)	80(83.3)	0,093^¥^
Betabloqueador, n (%)	98(89.1)	94(97,9)	0,076^¥^
BNP (pg/ml)	382,5±522,0	461,9±447,9	0,413*

^¥^teste qui-quadrado; *Teste t independente. Dados categóricos e contínuos foram apresentados. P<0,05 valor que foi considerado estatisticamente significativo. IECA: inibidor da enzima conversora de angiotensina; BRA: bloqueador do receptor de angiotensina; BNP: peptídeo natriurético cerebral; DCIC: doença cardíaca isquêmica crônica; DRC: doença renal crônica; DPOC: doença pulmonar obstrutiva crônica; DM: diabetes mellitus; HL: hiperlipidemia; NYHA CF: Classe Funcional da New York Heart Association.

Os parâmetros de monitoramento ambulatorial da PA não apresentaram diferenças significativas entre os grupos. A Tabela 2 mostra uma comparação dos achados da MAPA de 24 horas nos grupos de estudo.

**Tabela 2:** – Valores da pressão arterial nos casos dipper e não-dipper

	Dipper (n:110, mmHg)	Não-dipper (n:96, mmHg)	valor-p*
PAS Consultório	127,6±16,1	122,6±20,0	0,164
PAD Consultório	72,6±8,9	70,2±10,5	0,209
PAS 24h	128,3±10,6	128,9±13,7	0,748
PAD 24h	78,4±7,4	75,7±7,4	0,067
PAS diurna	130,9±11,7	132,8±14,0	0,472
PAD diurna	81,2±8,2	78,2±7,9	0,065
PAS noturna	113,8±13,6	115,4±15,5	0,578
PAD noturna	72,3±8,4	69,8±8,4	0,137
PPAM	31,1±6,7	32,9±6,3	0,174

* Teste t independente. PAD: pressão arterial diastólica; PPAM: aumento matinal da pressão arterial; PAS: pressão arterial sistólica. P<0,05 valor que foi considerado estatisticamente significativo. Dados contínuos foram apresentados.

Uma comparação dos achados ecocardiográficos nos grupos de estudo é apresentada na Tabela 3. O grupo não-dipper apresentou E/VP significativamente maior (p<0,001), relação E/Em (p=0,001) e índice de volume do átrio esquerdo (p<0,001) em comparação com o grupo dipper.

**Tabela 3:** – Achados ecocardiográficos em casos dipper e não-dipper

	Dipper (n:110)	Não-dipper (n:96)	valor-p*
IVAE (ml/m^2^)	33,1±4,2	38,1±5,7	**<0,001**
FEVE (%)	30,4±5,4	32,0±6,1	0,155
DVED (cm)	6,12±0,59	5,97±0,64	0,232
DVE (cm)	5,16±0,63	5,21±0,71	0,692
E (cm/s)	102,8±28,2	116,2±33,3	**0,029**
A (cm/s)	87,7±22,9	88,4±28,6	0,881
Relação E/A	1,26±0,51	1,57±0,91	0,061
TD (ms)	167,2±44,3	159,6±42,2	0,374
VP (cm/s)	37,4±4,2	32,2±4,4	**<0,001**
IVRT (ms)	132,7±26,1	142,5±43,4	0,164
Em (cm/s)	7,65±2,12	6,30±1,84	**0,001**
Am (cm/s)	7,12±1,67	7,73±2,64	0,160
Relação E/Em	11,8±4,4	16,4±7,8	**0,001**
E/VP	2,76±0,75	3,75±1,20	**<0,001**
Sm (cm/s)	6,84±1,07	6,41±1,36	0,082

*Teste t independente. A: pico tardio da velocidade de entrada mitral; Am: velocidade diastólica tardia do anel mitral; TD: tempo de desaceleração; E: pico precoce da velocidade de influxo mitral; Em: velocidade diastólica inicial do anel mitral; Relação E/A: relação entre o pico precoce (E) e o tardio (A) da velocidade de influxo mitral, E/Em: relação entre o pico inicial (E) da velocidade de influxo mitral e a velocidade diastólica precoce do anel mitral (Em); IVRT: isovolumétrico tempo de relaxação; IVAE: índice de volume do átrio esquerdo; FEVE: fração de ejeção do ventrículo esquerdo; DVE: diâmetro diastólico final do ventrículo esquerdo; DVED: diâmetro sistólico final do ventrículo esquerdo; Sm: velocidade sistólica Doppler tissular de pico; VP: velocidade de propagação do fluxo. P<0,05 valor que foi considerado estatisticamente significativo. Dados contínuos foram apresentados.

### Correlações

Houve correlações positivas entre PPAM e índice de volume do átrio esquerdo, tempo de desaceleração, E/VP e E/Em, e correlações negativas entre PPAM e VP e Em. A correlação significativa dos parâmetros com o PPAM também se manteve estatisticamente significativa quando controlada para fatores de confusão (idade e sexo) (Tabela 4).

**Tabela 4:** – Análise de correlação de PPAM e achados ecocardiográficos e parâmetros de MAPA de 24 horas

	PPAM		PPAM (ajustado por idade/sexo)	

	R	valor-p	R	valor-p
Gênero	0,147	0,139		
Idade	0,011	0,914		
Tabagismo	0,073	0,466	0,102	0,309
PAS Consultório	0,013	0,894	-0,001	0,995
PAD Consultório	0,067	0,500	0,103	0,680
PAS 24h	0,140	0,157	0,148	0,139
PAD 24h	0,017	0,865	0,038	0,704
PAS diurna	0,182	0,066	0,190	0,057
PAD diurna	0,057	0,568	0,081	0,421
PAS noturna	0,054	0,585	0,049	0,623
PAD noturna	-0,087	0,383	-0,081	0,422
IVAE	0,331	**0,001**	0,340	**0,001**
SIV	0,155	**0,035**	0,146	**0,048**
PP	0,201	**0,006**	0,201	**0,006**
IMVE	0,234	**0,001**	0,232	**0,002**
E	0,141	0,154	0,129	0,200
A	0,109	0,272	0,102	0,310
TD	0,268	**0,006**	0,275	**0,005**
VP	-0,357	**<0,001**	-0,353	**<0,001**
Relação E/A	0,026	0,794	0,014	0,887
Em	-0,338	**<0,001**	-0,328	**0,001**
Am	0,123	0,215	0,123	0,220
IVRT	0,045	0,652	0,060	0,548
Relação E/Em	0,314	**0,001**	0,300	**0,002**
Relação E/VP	0,326	**0,001**	0,314	**0,001**
BNP	0,008	0,933	-0,028	0,778

A: pico tardio da velocidade de entrada mitral; MAPA: monitoramento ambulatorial da pressão arterial; Am: velocidade diastólica tardia do anel mitral; PAD: pressão arterial diastólica; TD: tempo de desaceleração; E: pico precoce da velocidade de influxo mitral; Em: velocidade diastólica inicial do anel mitral; Relação E/A: relação entre o pico precoce (E) e o tardio (A) da velocidade de influxo mitral; E/Em: relação entre o pico inicial (E) da velocidade de influxo mitral e a velocidade diastólica precoce do anel mitral (Em); IVRT: isovolumétrico tempo de relaxar; SIV: septo interventricular; IVAE: índice de volume do átrio esquerdo; FEVE: fração de ejeção do ventrículo esquerdo; DVE: diâmetro diastólico final do ventrículo esquerdo; DVED: diâmetro sistólico final do ventrículo esquerdo; PPAM: aumento matinal da pressão arterial; PAS: pressão arterial sistólica; Sm: velocidade sistólica Doppler tissular de pico; IMVE: índice de massa ventricular esquerda; PP: parede posterior; VP: velocidade de propagação do fluxo. P<0,05 valor que foi considerado estatisticamente significativo.

### Análise da curva característica de operação do receptor

**Figura 1:**
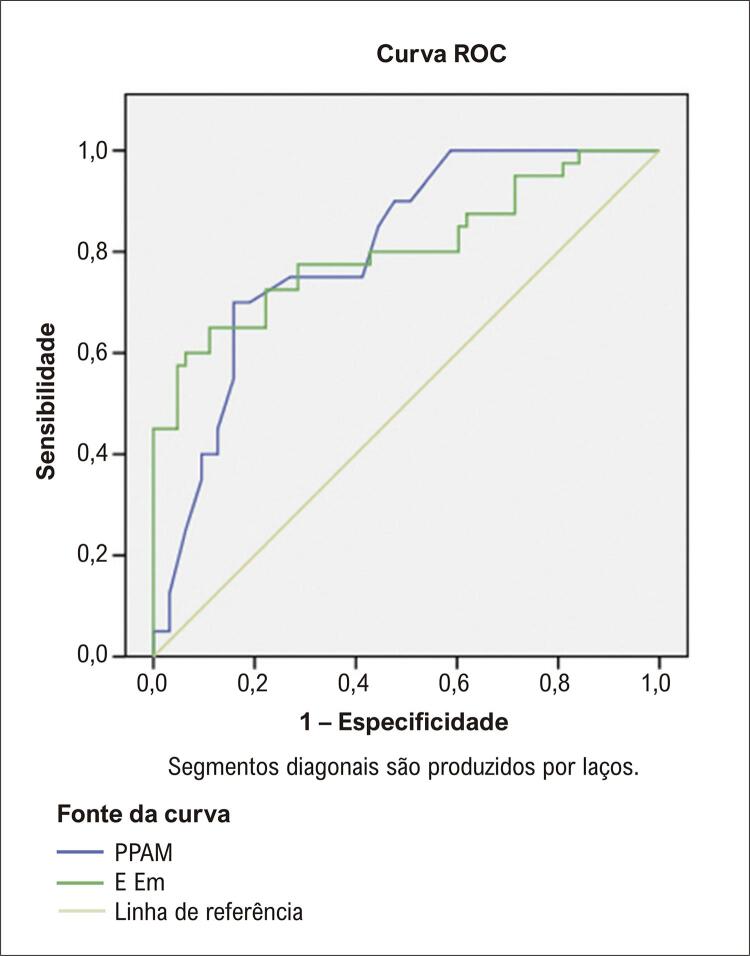
– Curva ROC (Receiver Operating Characteristic) para pico matinal de pressão arterial e E/Em na predição de internação; o valor de corte ideal para PPAM de 33,25 teve sensibilidade de 75,0% e especificidade de 73,0% área sob a curva característica de operação do receptor: 0,802, o valor de corte ideal para E/Em de 12,8 teve sensibilidade de 72,8% e especificidade de 74,6 % de área sob a curva característica de operação do receptor: 0,805.

A análise da curva característica de operação do receptor foi realizada para determinar os valores de corte de PPAM e E/Em para prever múltiplas internações hospitalares (Figura 1). O valor de corte para PPAM foi determinado em 33,25 mmHg e a área sob a curva foi de 0,802. Nesse valor de corte, a sensibilidade para predizer internações hospitalares foi de 75,0% e a especificidade foi de 73%. O valor de corte para E/Em foi de 12,8 e a área sob a curva foi de 0,805. Nesse valor de corte, a sensibilidade foi de 72,5% e a especificidade de 74,6%.

### Análise de Cox

Realizamos análise de Cox usando PPAM, padrão de PA sem imersão, E/Em, idade e sexo para prever internações hospitalares. A avaliação da relação entre essas variáveis independentes e múltiplas internações hospitalares mostrou que E/Em (razão de risco [RR] 1,04, p = 0,022), PPAM (RR 1,07, p = 0,016) e padrão de PA sem queda (RR 2,75, p=0,010) estiveram independentemente associados ao aumento de internações hospitalares (Tabela 5, Figura Central).

**Tabela 5:** – Análises de Cox entre taxa de internação hospitalar e idade, sexo, padrão de PA não-dipper, PPAM e E/Em

Variáveis independentes	RH	valor-p	IC de 95%
Idade	1.024	0,082	0,997-1,052
Género masculino	1.658	0,173	0,802-3,428
PPAM	1.071	**0,016**	1.013-1.133
PA não dipper	2.756	**0,010**	1.270-5.979
E/Em	1.049	**0,022**	1.007-1.092

PA: pressão arterial; E/Em: razão entre o pico inicial (E) da velocidade de influxo mitral e a velocidade diastólica precoce do anel mitral (Em); PPAM: aumento matinal da pressão arterial. P<0,05 valor que foi considerado estatisticamente significativo.

Também realizamos análise multivariada de Cox e descobrimos que idade (RR 1,14, p=0,014), NYHA FC (RR 5,25, p=0,019), FEVE (RR 0,64, p<0,001) e E/Em (RR 0,91, p= 0,036) foram preditores significativos de mortalidade (Tabela 6).

**Tabela 6:** – Preditores de Mortalidade na População com Insuficiência Cardíaca

Variáveis independentes	RH	valor-p	IC de 95%
Idade	1,146	0,014	1.028-1.277
Genero masculino	5,227	0,078	0,831-32,888
PPAM	1,028	0,690	0,898-1,177
PA não-dipper	1,356	0,639	0,379-4,853
E/Em	0,918	**0,039**	0,847-0,996
FEVE	0,649	**<0,001**	0,521-0,809
**NYHA FC**		**0,039**	
III contra II	3,225	0,112	0,535-19,441
IV contra II	5,252	**0,019**	1.679-38.861
BNP	1,000	0,917	0,999-1,001

PA: pressão arterial; BNP: peptídeo natriurético cerebral; E/Em: razão entre o pico inicial (E) da velocidade de influxo mitral e a velocidade diastólica inicial do anel mitral (Em); FEVE: fração de ejeção ventricular esquerda; PPAM: aumento matinal da pressão arterial; NYHA FC: Classe Funcional da New York Heart Association. P<0,05 valor que foi considerado estatisticamente significativo.

## Discussão

A relação entre HA e insuficiência cardíaca está se tornando uma questão clínica cada vez mais importante porque a coexistência das duas condições é frequente e, de acordo com dois grandes registros, a prevalência de HA aumentou de 47% para 59% nos últimos 10 anos.^14,15^ Em seu estudo, Yancy et al. especularam que 69% dos pacientes com ICFEr tinham PA elevada.^16^ Há quem sugira que a HA cause insuficiência cardíaca,^15^ bem como aqueles que sugerem que a presença de HA é uma comorbidade ou condição contribuinte em pacientes com insuficiência cardíaca.^17^ Em ambos os cenários, o controle da PA deve ser um dos principais objetivos em pacientes com insuficiência cardíaca. Embora muitos estudos tenham mostrado que características de PA não-dipper e PPAM estão associadas a maiores complicações cardiovasculares e danos a órgãos-alvo,^18,19^ não temos conhecimento de nenhum dado publicado sobre a relação entre ICFEr e achados de MAPA de 24 horas no contexto de PA noturna e PPAM.

Nosso estudo avaliou a variabilidade da PA em pacientes com ICFEr devido à alta taxa de comorbidade de HA. Em nosso estudo, o grupo não-dipper apresentou taxas significativamente maiores de internação hospitalar, uso de diuréticos, IVAE, E/Em e E/VP, de acordo com a literatura.^20^ A avaliação prognóstica de pacientes com insuficiência cardíaca grave é importante por causa disso alta morbimortalidade do grupo. Portanto, muitos métodos, como ecocardiografia, ressonância magnética cardíaca, MAPA de 24 horas, técnicas nucleares, cateterismo cardíaco, cintilografia de perfusão miocárdica e parâmetros laboratoriais, têm sido sugeridos para prever o prognóstico. Em um estudo retrospectivo, Cruz et al. especularam que um programa de telemonitoramento não invasivo reduziu significativamente as hospitalizações por IC e as admissões em departamentos de emergência.^21^ Muitos estudos mostram que os achados da MAPA de 24 horas se correlacionam com o prognóstico de pacientes com insuficiência cardíaca congestiva.^22-24^ O padrão de PA non-dipper é um desses fatores prognósticos.

Muitos possíveis mecanismos fisiopatológicos entre não-dipping e insuficiência cardíaca congestiva (ICC) foram sugeridos. Primeiro, o aumento da PA noturna tem sido associado ao comprometimento do enchimento ventricular esquerdo.^25^ Em segundo lugar, o padrão de PA não-dipper está associado à disfunção endotelial, que tem um papel principal na fisiopatologia da ICC. Higashi et al. descobriram que a disfunção endotelial estava associada à progressão e prognóstico da ICC.^26^ Em terceiro lugar, o padrão de PA não-dipper está associado ao aumento da atividade simpática, outro fator que se acredita estar envolvido na fisiopatologia da ICC.

Canesin et al. especularam que a PA sistólica mais baixa e o maior declínio noturno da PA no MAPA de 24 horas foram preditores de maior mortalidade em pacientes com insuficiência cardíaca sistólica avançada.^27^ Em outro estudo, Kastrup et al. compararam 25 pacientes com insuficiência cardíaca sistólica (grupos dipper e não-dipper) e 25 controles saudáveis. Eles determinaram que o padrão não-dipper era mais frequente em pacientes com insuficiência cardíaca sistólica em comparação com o grupo de controle saudável e sugeriram que pode ser mais prejudicial nesse grupo de pacientes.^28^ Neste estudo, também determinamos que a PA não-dipper padrão é mais prejudicial e que o comprometimento diastólico foi mais proeminente no grupo não-dipper, consistente com os achados relatados por Kastrup et al. Além disso, não encontramos uma relação significativa entre padrão não-dipper, PPAM e mortalidade. Vários fatores causam mortalidade em pacientes com insuficiência cardíaca, e a variabilidade da pressão arterial é apenas um desses fatores de risco. Em um estudo de amostra tão pequena, as características dos pacientes, as comorbidades e o uso de múltiplos medicamentos podem causar resultados insignificantes, contrários às expectativas de fatores de risco, como padrão não-dipper e PPAM.

Em contraste, Ueda et al. especularam que o padrão ascendente estava associado a um risco aumentado de desfechos adversos entre pacientes com ICFEp, mas não em pacientes com ICFEr.^29^ Em consonância com Ueda et al., Komori et al. demonstraram que pacientes com ICFEp com padrão de PA ascendente tinham um risco maior de resultado composto do que os pacientes dipper, mas essa relação não foi observada no grupo ICFEr.^30^ Em nosso estudo, não houve relação significativa entre PPAM, padrão não-dipper, e mortalidade entre pacientes com ICFEr, mas as internações hospitalares foram associadas ao padrão não-dipper e PPAM. Em outro estudo, Moroni et al. sugeriram que o ritmo circadiano da PA estava preservado em pacientes com insuficiência cardíaca sistólica.^31^ Ao contrário de nossos resultados, Kotti et al. observaram que a PA sistólica mais baixa e a queda no MAPA de 24 horas predisseram maior mortalidade.^24^ Em outro estudo, Shin et al. recrutaram 118 pacientes com insuficiência cardíaca sistólica e os acompanharam por 4 anos. Os pacientes foram divididos em grupos dipper, não-dipper e reverse dipper com base nos resultados do MAPA de 24 horas. Eles descobriram que as taxas de resultados adversos foram mais altas no grupo dipper reverso.^32^

Achados discrepantes têm sido relatados na literatura sobre a relação entre alterações circadianas da pressão arterial e desfechos cardiovasculares em pacientes com ICFEr e ICFEp, conforme mencionado acima. As razões para essas diferenças podem ser que (i) devido aos diferentes históricos dos pacientes, comorbidades e medicamentos, a importância da sensibilidade do barorreflexo pode diferir entre pacientes com ICFEr e ICFEp. (ii) O efeito adverso do PPAM pode ser mais proeminente em pacientes com ICFEr, reduzindo o volume sistólico. (iii) Essa discrepância pode depender da extensão e duração da elevação da PA. Embora a elevação transitória da PA, como o PPAM, possa exacerbar os resultados na ICFEr, o efeito da elevação duradoura da PA, como o padrão ascendente, pode ser insignificante em comparação com a ICFEp.^33^Definição e medição mais padronizadas para mudanças na pressão arterial circadiana são críticas para reconciliar essas descobertas aparentemente discrepantes.

A relação entre PPAM e prognóstico em pacientes com insuficiência cardíaca tornou-se uma questão clínica interessante nos últimos anos, pois piores desfechos foram associados a valores mais altos de PPAM.^34^ Komori et al. mostraram que o PPAM foi associado a um pior prognóstico em pacientes com insuficiência cardíaca com fração de ejeção reduzida.^33^ Em consonância com Komori et al., descobrimos que maior PPAM foi associado a mais internações hospitalares em pacientes com insuficiência cardíaca. O papel negativo da disfunção diastólica na formação da insuficiência cardíaca está fora de dúvida agora. Até o momento, até o momento, nenhum estudo na literatura tentou avaliar a relação entre PPAM e disfunção diastólica em pacientes com ICFEr. Benfari et al. demonstraram que a disfunção diastólica estava associada a pior IC, independente de todas as características de apresentação.^35^ Em consonância com Benfari et al., descobrimos que o comprometimento diastólico estava associado a piores desfechos de IC e descobrimos que os níveis de PPAM estavam associados à disfunção diastólica e IMVE em pacientes com insuficiência cardíaca sistólica. Em nosso estudo, diferentemente de outros achados do MAPA, observamos uma correlação entre PPAM e aumento de internações, mas não mortalidade, e observamos que a incidência de internações foi significativamente maior em pacientes com valores maiores de PPAM, especialmente acima de 33,25 mmHg. Se este valor de corte for confirmado em estudos futuros, pode ser útil na prática clínica para predizer internações hospitalares. A base fisiológica do aumento do PPAM é complexa e parece envolver muitos mecanismos interconectados. Possíveis mecanismos responsáveis pelo PPAM incluem ativação súbita do sistema simpático, diminuição da sensibilidade do barorreflexo e sistema renina-angiotensina-aldosterona. As causas do PPAM em pacientes com IC permanecem desconhecidas. Sensibilidade barorreflexa prejudicada foi observada em pacientes com IC, levando à ativação do sistema nervoso simpático. Esse mecanismo pode ser a principal causa do PPAM. Além disso, o PPAM aumenta a pós-carga cardíaca e a rigidez arterial, contribuindo para a progressão da hipertrofia e disfunção diastólica do VE. A insuficiência cardíaca congestiva também está relacionada às alterações citadas, que irão influenciar diretamente na PA matinal, pois a insuficiência cardíaca sistólica altera a variação circadiana normal da PA. Assim, pode-se especular que a insuficiência cardíaca pode induzir as vias que causam PPAM. A forte relação entre PPAM e morbidade cardiovascular neste estudo sugere que o PPAM pode ser um efeito secundário e indicador de insuficiência cardíaca em vez de uma causa direta de internações hospitalares ou que o PPAM pode ter aumentado as internações hospitalares alterando a função diastólica ou contribuindo para comprometimento diastólico.

Nosso estudo descobriu que padrões de PA non-dipper e PPAM foram fatores de risco independentes para internações hospitalares. Em seu estudo, Pierdomenico et al. mostraram que o maior risco cardiovascular entre pacientes hipertensos idosos tratados foi observado entre os dippers extremos com alto PPAM, seguidos por dippers reversos, não-dippers e dippers com alto PPAM. Além disso, eles mostraram que os dippers apenas com alto PPAM e os não-dippers estavam associados ao risco cardiovascular.^36^ Embora o desenho e a população do estudo de Pierdomenico não reflitam totalmente nosso estudo, a avaliação de seus resultados com os nossos destaca que os padrões não-dipper e o PPAM podem afetar os resultados cardiovasculares independentemente. Além disso, nós exibimos diferenças significativas entre dippers e não-dippers em relação ao PPAM. Nossos resultados se alinham com os de Tutal et al., que descobriram que o PPAM não era significativamente diferente entre os não-dippers e os dippers.^37^

Nosso estudo tem algumas limitações. Em primeiro lugar, foi realizado um estudo retrospectivo em um único centro com uma amostra relativamente pequena. Como o cálculo rotineiro de PPAM para cada paciente submetido à avaliação de MAPA de 24 horas em nossa clínica começou no final de 2018, o tamanho da amostra do estudo é pequeno e o tempo de acompanhamento é curto. A segunda limitação importante é que nosso estudo não tinha um grupo de controle saudável. Uma comparação tripla incluindo mais pacientes e um grupo de indivíduos saudáveis minimizaria o erro alfa tipo 1. Em terceiro lugar, a generalização deste estudo é limitada porque incluímos indivíduos nas classes NYHA II, III e IV e não incluímos pacientes com classe NYHA 1. Em quarto lugar, não temos dados sobre o momento da administração do medicamento, o que poderia afetar a pressão arterial medidas e o ritmo circadiano. Quinto, a falta de significância estatística de vários fatores de risco suspeitos, como padrão non-dipper e PPAM para mortalidade em pacientes com IC, não deve ser interpretada como significando que esses não são possíveis fatores de risco em pacientes individuais.

## Conclusão

Como a coexistência de disfunção diastólica e HA é comum entre pacientes com ICFEr, o tratamento da disfunção diastólica e da HA em termos de PPAM e estado dipping, um de seus fatores de risco modificáveis, deve ser o principal foco terapêutico para reduzir as internações hospitalares. No presente estudo, observamos uma relação significativa entre PPAM, padrão de PA não-dipping, E/Em e aumento de internações hospitalares. Portanto, o PPAM deve estar entre os novos preditores de internações e um foco terapêutico junto aos já estabelecidos em pacientes com ICFEr. A redução do PPAM poderia, portanto, ser um novo alvo terapêutico para prevenir internações hospitalares por insuficiência cardíaca em pacientes hipertensos. Mais estudos são necessários para determinar seu valor clínico.
