# Supplementary material for: A Relação entre o Estado Dipping Noturno, Aumento da Pressão Arterial Matinal e Internações Hospitalares em Pacientes com a Insuficiência Cardíaca Sistólica
Source: Arq Bras Cardiol. 2023 Sep 4;120(9):e20220932. [Article in Portuguese] doi: 10.36660/abc.20220932 (PMC10519354; doi:10.36660/abc.20220932)
Supplement: Translation

# Translation: The Relationship between Nocturnal Dipping Status, Morning Blood Pressure Surge, and Hospital Admissions in Patients with Systolic Heart Failure

**Authors:** Ümmü Taş, Sedat Taş, Efe Edem

**Affiliations:** 1 Turkey Izmir Demokrasi Universitesi – Cardiology, Karabaglar – Turkey; 2 Turkey Manisa Celal Bayar University – Cardiology, Manisa – Turkey; 3 Turkey İzmir Tınaztepe University – Cardiology, İzmir – Turkey

**Keywords:** Blood Pressure Monitoring, Ambulatory, Arterial Pressure, Heart Failure, Hospitalization

## Abstract

**Background:**

Hypertension is a known risk factor for developing heart failure. However, there is limited data to investigate the association between morning blood pressure surge (MBPS), dipping status, echocardiographic parameters, and hospital admissions in patients with systolic heart failure.

**Objectives:**

To evaluate the relationship between morning blood pressure surge, non-dipper blood pressure pattern, echocardiographic parameters, and hospital admissions in patients with systolic heart failure.

**Methods:**

We retrospectively analyzed data from 206 consecutive patients with hypertension and a left ventricular ejection fraction below 40%. We divided the patients into two groups according to 24-hour ambulatory blood pressure monitoring (ABPM) results: dippers (n=110) and non-dippers (n=96). Morning blood pressure surge was calculated. Echocardiographic findings and hospital admissions during follow-up were noted. Statistical significance was defined as p < 0.05.

**Results:**

The study group comprised 206 patients with a male predominance and mean age of 63.5 ± 16.1 years. The non-dipper group had significantly more hospital admissions compared to dippers. There was a positive correlation between MBPS and left atrial volume index (r=0.331, p=0.001), the ratio between early mitral inflow velocity and flow propagation velocity (r= 0.326, p=0.001), and the ratio between early mitral inflow velocity and mitral annular early diastolic velocity (E/Em) (r= 0.314, p=0.001). Non-dipper BP, MBPS, and E/Em pattern were found to be independently associated with increased hospital admissions.

**Conclusion:**

MBPS is associated with diastolic dysfunction and may be a sensitive predictor of hospital admission in patients with systolic heart failure.

**Central Illustration:** : The relationship between nocturnal dipping status, morning blood pressure and hospital admissions in patients with systolic heart failure

BP: Blood pressure; MBPS: Morning blood pressure surge; E/Em: Radio of early (E) peak of mitral inflow velocity to early (Em) diastolic mitral annular velocity.

## Introduction

Hypertension (HT) is among the most treatable cardiovascular disease risk factors. Observations regarding circadian variations in BP led to a new classification in HT. O’Brien et al. drew attention to the prognostic significance of the night-time reduction in blood pressure (BP) and first proposed the ‘dippers’ and ‘non-dippers’ concepts.^1^ Dippers were defined as people whose night-time BP decreased by 10% or more than day-time values, while those whose night-time BP decreased less than 10% are non-dippers. As BP already decreases at night with the normal circadian rhythm, the reduction of nocturnal BP in HT patients is defined as a good prognostic factor.^2^ Thus, 24-hour ambulatory blood pressure monitoring (24-h ABPM) to identify dipping or non-dipping patterns has become increasingly important for managing patients with HT.

Cardiovascular parameters like BP, coronary tone, and heart rate change with circadian rhythm.^3^ Fox and Mulcahy showed that circadian variations in heart rate and BP were virtually identical in normotensive subjects; both fell and remained relatively low throughout the night and then rose sharply in the early morning hours to reach a peak during the morning.^4^ There is growing interest in the role of non-dipping HT and the morning BP surge (MBPS) in various cardiovascular and cerebrovascular diseases, including left and right ventricular diastolic dysfunction, left ventricular hypertrophy, myocardial infarction, heart failure, and stroke.^5,6^ The influence of arterial hypertension on left ventricular structural and functional remodeling is well known.^7,8^ However, the effects of MBPS and non-dipper pattern on diastolic function and hospital admissions in patients with heart failure with reduced ejection fraction (HFrEF) are poorly described.

Heart failure is an important public health problem due to its association with significant morbidity, recurrent hospital admissions, increased healthcare costs, and mortality.^9,10^In their study, Mosalpuria et al. analyzed data from outpatient visits of heart failure patients. They found HT as the most common comorbidity (62%) and suggested that better outpatient management could improve outcomes and reduce the number of rehospitalizations.^11^ Evaluating the role of circadian BP variation in heart failure may shed light on the effectiveness of treatment and the timing of medication dosing in individual cases. Hospital admission, especially recurrent, due to heart failure is an important risk factor for mortality. Therefore, determining risk factors of hospital admission and preventing them may be a considerable and cost-effective method to reduce mortality due to HF. This study was conducted to determine the effects of circadian BP variability on hospital admission in patients with HFrEF and analyze the relationships between the nocturnal decline in BP, MBPS, and hospital admission.

## Methods

### Study populations

This retrospective case-control study was designed and conducted following the Declaration of Helsinki and approved by an institutional ethics committee of İzmir Tınaztepe University (Date and decision number: 2021/13). We collected the ABPM and echocardiography findings of 224 patients with systolic heart failure who presented to the cardiology outpatient clinics of our hospital between November 2018 and December 2019 and were previously diagnosed or newly diagnosed HT based on the outpatient clinic initial evaluation and then performed 24-hour ambulatory blood pressure monitoring. We also collected hospital admissions of each patient from the initial evaluation to 2 years follow-up. Of the 224 patients, we excluded those lost to follow-up (n = 18). Data regarding the patient’s hospital admissions during follow-up, medical history, and sociodemographic and clinical information were obtained from their medical records. Body mass index was noted for each patient (kg/m^2^).

We defined systolic and diastolic heart failure according to 2021 ESC guidelines for diagnosing and treating acute and chronic heart failure.^12,13^ HFrEF was defined as a left ventricular ejection fraction of less than 40%. Hospital admissions during follow-up were identified for all patients. We assessed all hospital admissions due to symptomatic heart failure 2 years after the initial evaluation.

### Ambulatory blood pressure monitoring

We defined HTas a systolic BP higher than 140 mmHg and/or diastolic BP higher than 90 mmHg, according to the criteria specified in the 2018 European Society of Hypertension/European Society of Cardiology (ESC) Guidelines^12^ for the management of arterial HT. For this study, we calculated day-time ambulatory BP as the average BP from 6 am to 8 pm and nocturnal BP as the average BP between 8 pm to 6 am. We also determined mean 24-h, day-time, and night-time systolic and diastolic BP values and calculated MBPS by subtracting the minimum night-time systolic BP from the mean systolic BP in the first 2 hours after getting up. Based on the results of 24-h ABPM, the patients were divided into the dipper group (≥10% decrease in night-time BP) and non-dippers (<10% decrease in night-time BP).

### Echocardiography

Left ventricular systolic and diastolic diameters, left atrial volume index, aortic diameter, left ventricular ejection fraction (Simpson’s method), mitral inflow E and A velocities, deceleration time, isovolumic relaxation time, the ratio of early (E) to late (A) peak of mitral inflow velocity, the ratio between early mitral inflow velocity and mitral annular early diastolic velocity (E/Em), and tissue Doppler findings assessed by two-dimensional echocardiography obtained from standard parasternal long- and short-axis and apical 2-, 3- and 4-chamber views were collected.

### Statistical analysis

The data were analyzed using SPSS version 15.0 (SPSS Inc, Chicago, USA). The normality test was performed by using the Kolmogorov-Smirnov test. Categorical data are expressed as count and percentage, and continuous data as mean and standard deviation (SD). Categorical data were compared using the chi-square test, and continuous data were compared using an independent t-test. Pearson correlation coefficients (r) were used to examine correlations between the MBPS and brain natriuretic peptide, BP values, and echocardiographic findings. A partial correlation was conducted to control the effect of the confounding factors, including gender and age. Factors with statistical significance (p <0.05) were included in logistic regression analysis to determine independent predictors of hospital admission. A variable selection procedure was implemented, and all these candidate variables were fed into the forward selection and backward elimination procedures, in addition to forcing all the variables in the model together. Multivariable Cox proportional hazards analyses using the Enter method were calculated to identify the independent predictors of mortality. Receiver operating characteristic curve analysis of selected variables was done to detect the cut-off values with the highest sensitivity and specificity in predicting hospital admissions. Statistical significance was defined as p < 0.05.

## Results

### Baseline and clinical characteristics

The study group comprised 206 patients with male predominance (84 [40.8%] women, 122 [59.2%] men) and a mean age of 63.5 ± 16.1 (24–94) years. According to the results of 24-h ABPM, 110 patients (53.3%) were dippers, and 96 patients (46.7%) were non-dippers. New York Heart Association (NYHA) class was II in 54.4%, III in 38.8%, and IV in 6.8% of the patients. During the study period, 206 patients had 556 hospital admissions due to confirmed heart failure (1.34 admissions per patient per year). The non-dipper group had a significantly higher hospital admission rate than the dipper group (p<0.001). There were no significant differences between the groups in comorbid diseases, including diabetes mellitus, chronic kidney disease, chronic ischemic heart disease, chronic obstructive pulmonary disease, and hyperlipidemia, or terms of the drugs used by the patients except diuretics. The clinical characteristics of the patients are summarized in Table 1.

**Table 1:** – Baseline clinical and demographic characteristics

	Dipper (n:110)	Non-dipper (n:96)	p-value
Gender, male(%)	68(61.8)	54(56.3)	0.566^¥^
Age (year)	57.0±16.3	60.1±15.8	0.325*
**NYHA FC, n (%)**			0.094^¥^
FC-2	70(63.6)	42(43.8)	
FC-3	36(32.7)	44(45.8)	
FC-4	4(3.6)	10(10.4)	
The number of hospital admission (year)	1.01±0.59	1.72±0.81	<0.001^*^
Smoking, n(%)	16(14.5)	16(16.7)	0.767^¥^
DM, n(%)	34(30.9)	32(33.3)	0.793^¥^
CKD, n(%)	10(9.1)	10(10.4)	0.821^¥^
HL, n(%)	22(20.0)	24(25.0)	0.543^¥^
CIHD, n(%)	72(65.5)	74(77.1)	0.195^¥^
COPD, n(%)	6(5.5)	16(16.7)	0.066^¥^
Diuretic, n(%)	82(74.5)	94(94.9)	**0.001**^**¥**^
ACEI-ARB, n(%)	94(85.5)	84(87.5)	0.806^¥^
Spironalacton, n(%)	76(69.1)	80(83.3)	0.093^¥^
Beta-blocker, n(%)	98(89.1)	94(97.9)	0.076^¥^
BNP (pg/ml)	382.5±522.0	461.9±447.9	0.413*

^¥^Chi-square test; * Independent t-test. Categorical and continuous data were presented. P<0.05 value that was considered statistically significant. ACEI: angiotensin-converting enzyme inhibitor; ARB: angiotensin receptor blocker; BNP: brain natriuretic peptide; CIHD: chronic ischemic heart disease; CKD: chronic kidney disease; COPD: chronic obstructive pulmonary disease; DM: diabetes mellitus; HL: hyperlipidemia; NYHA FC: New York Heart Association Functional Class.

Ambulatory BP monitoring parameters showed no significant differences between the groups. Table 2 shows a comparison of 24-h ABPM findings in the study groups.

**Table 2:** – Blood pressure values in dipper and non-dipper cases

	Dipper (n:110, mmHg)	Non-dipper (n:96, mmHg)	p-value*
Office SBP	127.6±16.1	122.6±20.0	0.164
Office DBP	72.6±8.9	70.2±10.5	0.209
24-h SBP	128.3±10.6	128.9±13.7	0.748
24-h DBP	78.4±7.4	75.7±7.4	0.067
Day-time SBP	130.9±11.7	132.8±14.0	0.472
Day-time DBP	81.2±8.2	78.2±7.9	0.065
Night-time SBP	113.8±13.6	115.4±15.5	0.578
Night-time DBP	72.3±8.4	69.8±8.4	0.137
MBPS	31.1±6.7	32.9±6.3	0.174

*Independent t-test. DBP: diastolic blood pressure; MBPS: morning blood pressure surge; SBP: systolic blood pressure. P<0.05 value that was considered statistically significant Continuous data were presented.

A comparison of echocardiographic findings in the study groups is shown in Table 3. The non-dipper group had significantly higher E/VP (p<0.001), E/Em ratio (p=0.001), and left atrial volume index (p<0.001) compared to the dipper group.

**Table 3:** – Echocardiographic findings in dipper and non-dipper cases

	Dipper (n:110)	Non-dipper (n:96)	p-value*
LAVI (ml/m^2^)	33.1±4.2	38.1±5.7	**<0.001**
LVEF (%)	30.4±5.4	32.0±6.1	0.155
LVEDD (cm)	6.12±0.59	5.97±0.64	0.232
LVESD (cm)	5.16±0.63	5.21±0.71	0.692
E (cm/s)	102.8±28.2	116.2±33.3	**0.029**
A (cm/s)	87.7±22.9	88.4±28.6	0.881
E/A ratio	1.26±0.51	1.57±0.91	0.061
DT (ms)	167.2±44.3	159.6±42.2	0.374
VP (cm/s)	37.4±4.2	32.2±4.4	**<0.001**
IVRT (ms)	132.7±26.1	142.5±43.4	0.164
Em (cm/s)	7.65±2.12	6.30±1.84	**0.001**
Am (cm/s)	7.12±1.67	7.73±2.64	0.160
E/Em ratio	11.8±4.4	16.4±7.8	**0.001**
E/VP	2.76±0.75	3.75±1.20	**<0.001**
Sm (cm/s)	6.84±1.07	6.41±1.36	0.082

*Independent t-test. A: late peak of mitral inflow velocity; Am: late diastolic mitral annular velocity; DT: deceleration time; E: early peak of mitral inflow velocity; Em: early diastolic mitral annular velocity; E/A ratio: ratio of early (E) to late (A) peak of mitral inflow velocity; E/Em: ratio of early (E) peak of mitral inflow velocity to early (Em) diastolic mitral annular velocity; IVRT: isovolumic relaxation time; LAVI: left atrial volume index; LVEF: left ventricular ejection fraction; LVEDD: left ventricular end-diastolic diameter; LVESD: left ventricular end-systolic diameter; Sm: peak tissular Doppler systolic velocity; VP: flow propagation velocity. P<0.05 value that was considered statistically significant Continuous data were presented.

### Correlations

There were positive correlations between MBPS and left atrial volume index, deceleration time, E/VP, and E/Em, and negative correlations between MBPS and VP and Em. The significant correlation of parameters with MBPS also remained statistically significant when controlled for confounders (age and gender) (Table 4).

**Table 4:** – Correlation analysis of MBPS and echocardiographic findings and 24-h ABPM parameters

	MBPS		MBPS (adjusted for age/gender)	

	R	p-value	R	p-value
Gender	0.147	0.139		
Age	0.011	0.914		
Smoking	0.073	0.466	0.102	0.309
Office SBP	0.013	0.894	-0.001	0.995
Office DBP	0.067	0.500	0.103	0.680
24-h SBP	0.140	0.157	0.148	0.139
24-h DBP	0.017	0.865	0.038	0.704
Day-time SBP	0.182	0.066	0.190	0.057
Day-time DBP	0.057	0.568	0.081	0.421
Night-time SBP	0.054	0.585	0.049	0.623
Night-time DBP	-0.087	0.383	-0.081	0.422
LAVI	0.331	**0.001**	0.340	**0.001**
IVS	0.155	**0.035**	0.146	**0.048**
PW	0.201	**0.006**	0.201	**0.006**
LVMI	0.234	**0.001**	0.232	**0.002**
E	0.141	0.154	0.129	0.200
A	0.109	0.272	0.102	0.310
DT	0.268	**0.006**	0.275	**0.005**
VP	-0.357	**<0.001**	-0.353	**<0.001**
E/A ratio	0.026	0.794	0.014	0.887
Em	-0.338	**<0.001**	-0.328	**0.001**
Am	0.123	0.215	0.123	0.220
IVRT	0.045	0.652	0.060	0.548
E/Em ratio	0.314	**0.001**	0.300	**0.002**
E/VP ratio	0.326	**0.001**	0.314	**0.001**
BNP	0.008	0.933	-0.028	0.778

A: late peak of mitral inflow velocity; ABPM: ambulatory blood pressure monitoring; Am: late diastolic mitral annular velocity; DBP: diastolic blood pressure; DT: deceleration time; E: early peak of mitral inflow velocity; Em: early diastolic mitral annular velocity; E/A ratio: ratio of early (E) to late (A) peak of mitral inflow velocity; E/Em: ratio of early (E) peak of mitral inflow velocity to early (Em) diastolic mitral annular velocity; IVRT: isovolumic relaxation time; IVS: interventricular septum; LAVI: left atrial volume index; LVEF: left ventricular ejection fraction; LVEDD: left ventricular end-diastolic diameter; LVESD: left ventricular end-systolic diameter; MBPS: morning blood pressure surge; SBP: systolic blood pressure; Sm: peak tissular Doppler systolic velocity; LVMI: left ventricular mass index; PW: posterior Wall; VP: flow propagation velocity. P<0.05 value that was considered statistically significant.

### Receiver operating characteristic curve analysis

Receiver operating characteristic curve analysis was performed to determine MBPS and E/Em cut-off values to predict multiple hospital admissions (Figure 1). The cut-off value for MBPS was determined as 33.25 mmHg, and the area under the curve was 0.802. At this cut-off value, the sensitivity for predicting hospital admissions was 75.0%, and the specificity was 73%. The cut-off value for E/Em was 12.8, and the area under the curve was 0.805. At this cut-off value, the sensitivity was 72.5%, and the specificity was 74.6%.

**Figure 1:** – The receiver operating characteristic (ROC) curve for morning blood pressure surge and E/Em in the prediction of hospital admission; the optimal cut-off value for MBPS of 33.25 had sensitivity of 75.0% and specificity of 73.0% area under receiver operating characteristic curve: 0.802, the optimal cut-off value for E/Em of 12.8 had sensitivity of 72.8% and specificity of 74.6% area under receiver operating characteristic curve: 0.805.

### Cox analysis

We performed Cox analysis using MBPS, non-dipping BP pattern, E/Em, age, and gender to predict hospital admissions. Evaluation of the relationship between these independent variables and multiple hospital admissions showed that E/Em (hazard ratio [HR] 1.04, p=0.022), MBPS (HR 1.07, p=0.016), and non-dipping BP pattern (HR 2.75, p=0.010) were independently associated with increased hospital admissions (Table 5, Central Illustration).

**Table 5:** – Cox analyses between hospital admission rate and age, gender, non-dipper BP pattern, MBPS, and E/Em

Independent variables	HR	p value	95% CI
Age	1.024	0,082	0,997-1,052
Gender (male)	1.658	0,173	0,802-3,428
MBPS	1.071	**0,016**	1.013-1.133
Non-dipper BP	2.756	**0,010**	1.270-5.979
E/Em	1.049	**0,022**	1.007-1.092

BP: blood pressure; E/Em: Ratio of early (E) peak of mitral inflow velocity to early (Em) diastolic mitral annular velocity; MBPS: Morning blood pressure surge. P<0.05 value that was considered statistically significant

We also performed multivariate Cox analysis and found that age (HR 1.14, p=0.014), NYHA FC (HR 5.25, p=0.019), LVEF (HR 0.64, p<0.001), and E/Em (HR 0.91, p=0.036) were significant predictors of mortality (Table 6).

**Table 6:** – Predictors of Mortality in Heart Failure Population

Independent variables	HR	p value	95% CI
Age	1.146	0.014	1.028-1.277
Gender (male)	5.227	0.078	0.831-32.888
MBPS	1.028	0.690	0.898-1.177
Non-dipper BP	1.356	0.639	0.379-4.853
E/Em	0.918	**0.039**	0.847-0.996
LVEF	0.649	**<0.001**	0.521-0.809
**NYHA FC**		0.039	
III versus II	3.225	0.112	0.535-19.441
IV versus II	5.252	**0.019**	1.679-38.861
BNP	1.000	0.917	0.999-1.001

BP: blood pressure; BNP: brain natriuretic peptide; E/Em: ratio of early (E) peak of mitral inflow velocity to early (Em) diastolic mitral annular velocity; LVEF: left ventricular ejection fraction; MBPS: morning blood pressure surge; NYHA FC: New York Heart Association Functional Class. P<0.05 value that was considered statistically significant.

## Discussion

The relationship between HT and heart failure is becoming an increasingly important clinical question because the coexistence of the two conditions is frequent, and according to two large registries, the prevalence of HT increased from 47% to 59% over the last 10 years.^14,15^ In their study, Yancy et al. speculated that 69% of patients with HFrEF had elevated BP.^16^ There are those who suggest that HT causes heart failure^15^ as well as those who suggest that the presence of HT is a comorbid or contributing condition in patients with heart failure.^17^In either scenario, controlling BP should be one of the primary goals in heart failure patients. Although many studies have shown that non-dipper BP characteristics and MBPS are associated with higher cardiovascular complications and target organ damage,^18,19^ we are not aware of any published data regarding the relationship between HFrEF and 24-h ABPM findings in the context of nocturnal BP and MBPS.

Our study evaluated BP variability in patients with HFrEF because of the high rate of comorbid HT. In our study, the non-dipper group exhibited significantly higher hospital admission rates, diuretic usage, LAVI, E/Em, and E/VP, consistent with the literature.^20^ The prognostic evaluation of patients with severe heart failure is important because of this group’s high morbidity and mortality rate. Therefore, many methods, such as echocardiography, cardiac magnetic resonance imaging, 24-h ABPM, nuclear techniques, cardiac catheterization, myocardial perfusion scintigraphy, and laboratory parameters, have been suggested to predict prognosis. In a retrospective study, Cruz et al. speculated that a non-invasive telemonitoring program has significantly reduced HF hospitalizations and emergency department admissions.^21^ Many studies show that 24-h ABPM findings correlate with the prognosis of congestive heart failure patients.^22-24^The non-dipper BP pattern is one of these prognostic factors.

Many possible pathophysiological mechanisms between non-dipping and congestive heart failure (CHF) have been suggested. First, increased night-time BP has been linked to left ventricular filling impairment.^25^ Second, non-dipper BP pattern is associated with endothelial dysfunction, which has a principal role in the pathophysiology of CHF. Higashi et al. found that endothelial dysfunction was associated with CHF progression and prognosis.^26^ Third, the non-dipper BP pattern is associated with increased sympathetic activity, another factor believed to be involved in the pathophysiology of CHF.

Canesin et al. speculated that lower systolic BP and larger night-time decline in BP on 24-h ABPM were predictors of higher mortality in patients with advanced systolic heart failure.^27^ In another study, Kastrup et al. compared 25 patients with systolic heart failure (dipper and non-dipper groups) and 25 healthy controls. They determined that the non-dipper pattern was more frequent in patients with systolic heart failure compared to the healthy control group and suggested that it may be more harmful in this patient group.^28^In this study, we also determined that the non-dipper BP pattern is more harmful and that diastolic impairment was more prominent in the non-dipper group, consistent with the findings reported by Kastrup et al. Furthermore, we did not find a significant relationship between non-dipper pattern, MBPS, and mortality. Several factors cause mortality in heart failure patients, and blood pressure variability is only one of those risk factors. In such a small sample study, the patients’ characteristics, comorbidities, and multiple drug usage may cause insignificant results contrary to expectations for risk factors such as non-dipper pattern and MBPS.

In contrast, Ueda et al. speculated that the riser pattern was associated with an increased risk of adverse outcomes among patients with HFpEF but not in patients with HFrEF.^29^ In line with Ueda et al., Komori et al. exhibited that HFpEF patients with the riser BP pattern had a higher risk of composite outcome than the dipper patients, but this relationship was not seen in the HFrEF group.^30^ In our study, there was no significant relationship between MBPS, non-dipper pattern, and mortality among HFrEF patients, but the hospital admissions were associated with non-dipper pattern and MBPS. In another study, Moroni et al. suggested that the circadian rhythm of BP was preserved in patients with systolic heart failure.^31^ Unlike our results, Kotti et al. observed that lower systolic BP and dipping on 24-h ABPM predicted higher mortality.^24^ In another study, Shin et al. enrolled 118 patients with systolic heart failure and followed them for 4 years. The patients were divided into dipper, non-dipper, and reverse dipper groups based on 24-h ABPM results. They found that adverse outcome rates were highest in the reverse dipper group.^32^

Discrepant findings have been reported in the literature regarding the relationship between circadian blood pressure changes and cardiovascular outcomes in HFrEF and HFpEF patients, as mentioned above. The reasons for those differences could be that (i) because of the different patient backgrounds, comorbidities, and medication, the significance of baroreflex sensitivity could differ between patients with HFrEF and HFpEF. (ii) The adverse effect of MBPS may be more prominent in HFrEF patients by reducing stroke volume. (iii) This discrepancy could depend on the extent and duration of the BP elevation. While transient BP elevation, such as MBPS, could exacerbate outcomes in HFrEF, the effect of long-lasting BP elevation, such as riser pattern, may be negligible compared to HFpEF.^33^ More standardized definition and measurement for circadian blood pressure changes is critical to reconcile these seemingly discrepant findings.

The relationship between MBPS and prognosis in patients with heart failure has become an interesting clinical question in recent years, as worse outcomes have been associated with higher MBPS values.^34^ Komori et al. showed that MBPS was associated with a worse prognosis in patients with heart failure with a reduced ejection fraction.^33^ In line with Komori et al., we found that higher MBPS was associated with more hospital admissions in patients with heart failure. The negative role of diastolic dysfunction in the formation of heart failure is out of doubt now. To the best of our knowledge, no study in the literature has attempted to assess the relation between MBPS and diastolic dysfunction in patients with HFrEF up to now. Benfari et al. exhibited that diastolic dysfunction was associated with worse HF, independent of all presentation characteristics.^35^ In line with Benfari et al., we found that diastolic impairment was associated with worse outcomes of HF, and we also found that MBPS levels were associated with diastolic dysfunction and LVMI in patients with systolic heart failure. In our study, unlike other ABPM findings, we observed a correlation between MBPS and increased hospital admissions but not mortality, and we observed that the incidence of hospital admissions was significantly higher in patients with larger MBPS values, especially over 33.25 mmHg. If this cut-off value is confirmed in future studies, it may be useful in clinical practice for predicting hospital admissions. The physiological basis of increased MBPS is complex and seems to involve many interconnected mechanisms. Possible mechanisms responsible for MBPS include sudden sympathetic system activation, impaired baroreflex sensitivity, and the renin-angiotensin-aldosterone system. The causes of MBPS in HF patients remain unknown. Impaired baroreflex sensitivity was observed in HF patients, leading to sympathetic nervous system activation. This mechanism could be the main cause of MBPS. Furthermore, MBPS increases cardiac afterload and arterial stiffness, contributing to the progression of LV hypertrophy and diastolic dysfunction. Congestive heart failure is also related to the abovementioned alterations, which will directly influence morning BP because systolic heart failure alters the normal circadian variation in BP. Thus, it can be speculated that heart failure may induce the pathways causing MBPS. The strong relationship between MBPS and cardiovascular morbidity in this study suggests that MBPS may be a secondary effect and indicator of heart failure instead of a direct cause of hospital admissions or that MBPS may have increased hospital admissions by altering the diastolic function or contributing to diastolic impairment.

Our study found that non-dipper BP patterns and MBPS were independent risk factors for hospital admissions. In their study, Pierdomenico et al. exhibited that the highest cardiovascular risk among elderly-treated hypertensive patients was observed among the extreme dippers with high MBPS, followed by reverse dippers, non-dippers, and dippers with high MBPS. Furthermore, they showed that dippers only with high MBPS and non-dippers were associated with cardiovascular risk.^36^ Although Pierdomenico’s study design and population do not fully reflect our study, evaluating their results with ours highlights that non-dipper patterns and MBPS may affect cardiovascular outcomes independently. Furthermore, we exhibited no significant differences between dippers and non-dippers regarding MBPS. Our results align with those of Tutal et al., who found MBPS was not significantly different between non-dippers and dippers.^37^

Our study has some limitations. Firstly, a retrospective study was conducted with a relatively small sample at a single center. Since the routine calculation of MBPS for each patient who underwent 24-h ABPM evaluation in our clinic started at the end of 2018, the study’s sample size is small, and the follow-up time is short. The second important limitation is that our study lacked a healthy control group. A triple comparison including more patients and a group of healthy individuals would minimize the type-1 alpha error. Third, the generalizability of this study is limited because we included individuals in NYHA classes II, III, and IV and did not include patients with NYHA class 1. Fourth, we have no data on the timing of drug administration, which could affect blood pressure measurements and circadian rhythm. Fifth, the lack of statistical significance of several suspected risk factors, such as non-dipper pattern and MBPS for mortality in HF patients, should not be construed to mean that these are not possible risk factors in individual patients.

## Conclusion

As the coexistence of diastolic dysfunction and HT is common among HFrEF patients, treating diastolic dysfunction and HT in terms of MBPS and dipping status, one of its modifiable risk factors, should be a main therapeutic focus to reduce hospital admissions. In the present study, we observed a significant relationship between MBPS, non-dipping BP pattern, E/Em, and increased hospital admissions. Therefore, MBPS should be among the new predictors of hospital admissions and a therapeutic focus together with the established ones in patients with HFrEF. Reducing the MBPS could thus be a new therapeutic target for preventing hospital admissions due to heart failure in hypertensive patients. Further studies are needed to ascertain its clinical value.
